# Fatal Case of Popliteal Aneurism

**Published:** 1826-07

**Authors:** 


### POPLITEAL ANEURISM.*

On the 1st July, 1825, James Ferguson, a sailor, observed pain and swelling in the left ham; in ten days time the pain abated; the tumour remained and pulsated strongly. August 23d, he was admitted into the Royal Infirmary of Edinburgh. His symptoms were—the left leg semi-bent; motions of the knee-joint impaired; at times, shooting pains in the leg with œdema and tingling in the foot; in the ham was an elastic circumscribed tumour, projecting beyond the hamstrings and thrilling to the touch—the integuments above it tense, but not red or painful: the femoral artery pulsated strongly, the anterior and posterior tibial arteries indistinctly. Some of the lymphatic glands of the neck were enlarged, but his general health was good. He had not overstrained himself.

August 26th, the artery was tied in the usual manner, when the tension and pulsation of the tumour immediately ceased—he bore the operation well. At one o'clock, an hour after the operation, there was much pain in the wound and foot, which was a little colder than the other—8 o'clock, much the same, a bottle of hot water to the foot—anodyne. Aug. 27th, 7 A. M. he had slept well—there was no pain, merely a little numbness and tingling in the foot and toes; no pulsation

* Mr. Allan. Edinburgh Journ. of Med. Science, No. II. for April, 1826.

in the tumour, nor in the anterior or posterior tibial arteries; heat of the sound limb 94°, that of the other 88½. At noon the pulse was 96, full and strong; pain in the loins; no dejection; tongue clean—a cathartic enema was given immediately. In the evening he complained of pain in the leg. 28th, Nearly as before—an ounce of castor oil was given. 29th, The medicine had operated thrice—the wound was dressed—the edges had adhered—no tumefaction or inflammation. In the evening the pulse rose to 106, full and bounding; surface hot; V. S. ad ℥xiv. —Two aloetic pills to be taken in the morning. 30th, Better—pulse 104 and softer—a cathartic enema in the evening—after this there was little febrile action; the aneurismal limb was generally about two degrees colder than the sound one. September 4th, Costiveness; pain and starting in the leg; pulse 160 and sharp; wound surrounded with a blush of inflammation; adhesion of the edges perfect; inguinal glands enlarged and painful—saturnine lotion to the groin and wound: V. S. ad. ℥xiv, and two drachms of pulv. jalap. comp. to be taken immediately. September, The blood taken was sizy; better in all respects; the aneurismal tumour much reduced in size. Cold lotion and cathartic powder to be continued, and a saline mixture with antimonial wine to be taken occasionally. 6th, Erysipelas appeared on the thigh and haunch; infusion of senna with a little calomel at times was given, and it disappeared on the 13th. Severe pain now seized upon the knee, attended with some swelling and burning heat stretching down the calves of the legs. On the 18th, the ligature came away with little discharge—the wound cicatrized. From this to the 27th the pain, swelling, &c. of the knee continued; pulse 120; great febrile action and, at times, even delirium. He left the hospital on the 20th, for his father's house, the aneurismal tumour being about the size of a duck's egg. The treatment consisted of fomentations and poultices to the part, with the exhibition of diaphoretics and laxatives. September 27th, Distinct fluctuation round the joint; two incisions were made, one a little above the patella, whence issued six or seven ounces of bloody matter, the other two inches below the knee on the inner side, from which about an ounce of pus was discharged. Oiled lint was introduced into the openings, and poultices applied. For the next two days the discharge was profuse, and the irritative fever excessive. 30th, The limb was removed three inches below where the ligature was applied—eight vessels were secured—the femoral artery was observed to be open; it was tied, but, on taking off the tourniquet no pulsation could be perceived at the ligature—the stump was dressed in the usual way—the man went on well for three days; but then diarrhœa and night-sweats came on, and he sunk eight days after the operation.

*Dissection*. The popliteal vein lying over the tumour was obliterated, and filled with a clot of blood just before it came in contact with the lower part of the tumour—with regard to the aneurism itself, there was nothing remarkable—no abrasion of the internal coat of the artery could be perceived—there were three abscesses around the joint, communicating with one another, with the aneurismal sac, and with the joint, so that, in fact, the knee-joint was almost surrounded with pus which was also contained in its cavity—On examining the artery in the stump, it was found that the ligature had been applied three inches below the origin of the profunda; at this spot the vessel looked as if it had been divided transversely by a knife; the sheath of the artery, and cellular membrane around it were much thickened to the extent of an inch and a half above the ligature, and two inches below it; the cavity of the artery, from an inch above the ligature to the face of the stump was much contracted and filled with a clot.

*Remarks.* The ligature did not, it appears, come away till the 24th day—this was owing, in a great measure, to the state of the constitution, but partly also to the thread not having been drawn very tight. With regard to the patient's death, it is clear that it "was not to be imputed to the suppuration of the sac," for the operation, as far as regarded the aneurism was successful, "but to distinct abscesses, which, by inducing ulcerative absorption had formed a communication both with the joint and the aneurismal sac."

---

